# Necrotizing fasciitis secondary to Wiskott-Aldrich Syndrome: a unique clinical presentation. Case report

**DOI:** 10.1080/23320885.2025.2527095

**Published:** 2025-07-07

**Authors:** Jose Ignacio Fonseca-Sada, Roberto Martinez-Mejorada, Gabriel Garcia-Gonzalez, Luis Carlos Lozano-Carrillo, Rodolfo Alfredo Valdez-Velez, Mauricio Garcia-Perez, Everardo Valdes-Flores

**Affiliations:** Plastic, Aesthetic and Reconstructive Surgery Department, University Hospital “Dr. Jose Eleuterio Gonzalez”, Monterrey, Mexico

**Keywords:** Wiskott-Aldrich Syndrome, case report, necrotizing fasciitis, loxosceles reclusa

## Abstract

Wiskott-Aldrich Syndrome presents unique diagnostic and therapeutic challenges. Our case highlights a rare clinical complication associated with WAS and emphasizes the importance of prompt recognition and management. Dissemination of such rare presentations is crucial for enhancing clinical awareness and optimizing patient outcomes.

## 1. Introduction

Wiskott-Aldrich Syndrome (WAS) is a rare primary immunodeficiency disorder inherited in an X chromosome-linked pattern, meaning it is passed on by female carriers and primarily affects their male offspring. The disorder results from mutations in the WAS gene, which encodes the Wiskott-Aldrich syndrome protein (WASP), expressing exclusively in non-erythroid hematopoietic cells [1].

The estimated incidence is 3.7–4.1 per million live births [2,3]. The average survival in WAS patients is 20 years [4]. Typical clinical manifestations include thrombocytopenia, eczema, immunodeficiencies, as well as an increased risk of neoplasms and autoimmune manifestations [5]. Moderate to severe eczema has been described and requires continued treatment with emollients and topical steroids.

WASP dysfunction is responsible for the clinical manifestations due to the negative effects in several systems such as hematologic and immune manifesting as autoimmune haemolytic anaemia, arthritis, inflammatory bowel disease (IBD), vasculitis, lymphoma, among many other examples [6,7]. This case is notable due to its atypical presentation and evolution, which have been rarely documented in the literature.

## 2. Case presentation

We present the case of a 2-year-old male patient, the fourth son of non-consanguineous parents, native of Mexico; who presented at the Emergency Department with Septic Shock necessitating hospitalisation in the Intensive Care Unit and administration of vasopressors. Relevant clinical history includes a previous hospitalization 4 months before the current one for soft tissue cellulitis where *Haemophilus influenzae* was isolated; the infection progressed into a sepsis and AKI-I that was treated with antibiotics.

Six days into hospitalisation, the Plastic, Aesthetic, and Reconstructive Department was consulted due to ischemic changes manifested as a violet discolouration of the left lower extremity, with a presumptive diagnosis of a Loxocelism by Sicariidae, commonly known as a Brown Recluse Spider (Loxosceles reclusa) bite. The initial diagnosis was established by considering the clinical attributes of the lesions, coupled with the understanding that the spider in question is indigenous to our locality and is renowned for prompting visits to the emergency department due to its venomous bites.

**Figure 1.**
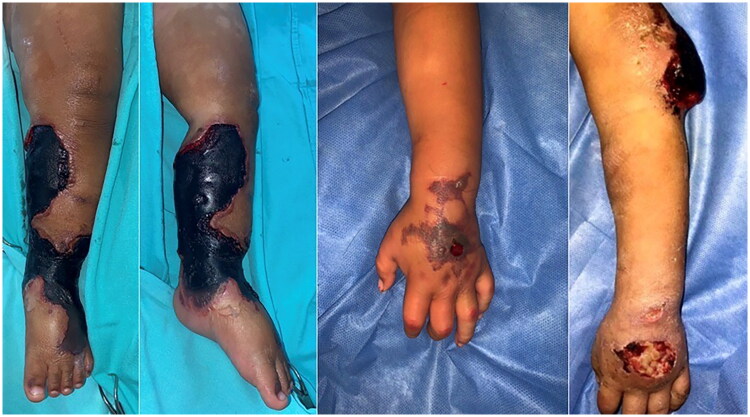
Necrotizing lesions in lower and upper left extremities.

**Figure 2.**
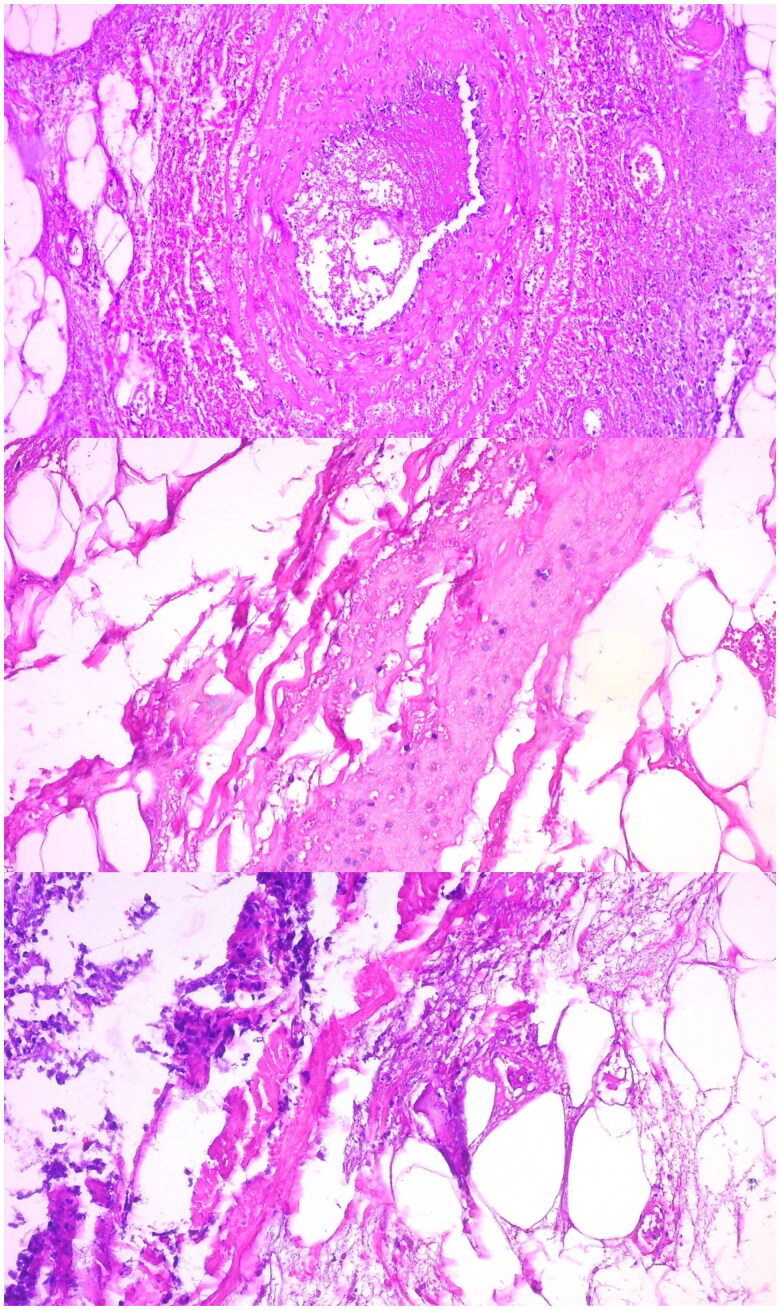
Pathology sample of the skin lesion revealing ischemic necrosis and suppurative panniculitis associated with microvascular thrombosis.

Two days later, the lesion showed signs of necrosis (see Figure 1). The patient underwent necrotic bedsore debridement and required skin grafts in the Operating Room (see Figure 2). Samples were forwarded to the pathology department, which reported the isolation of *Haemophilus influenzae* and *Staphylococcus aureus*.

No complications were reported, and the patient was discharged after two additional weeks of hospitalization due to clinical improvement. However, three months later, the patient developed a new purpuric lesion on the arm and left hand (see Figure 1), associated with intermittent fever peaks, rapidly progressing to a necrotizing lesion.

**Figure 3.**
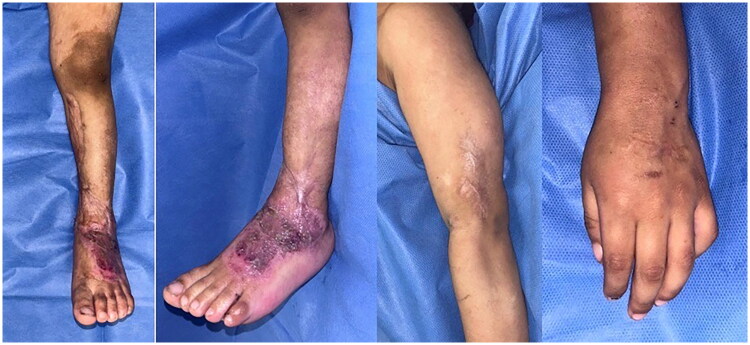
(A) Debridement of necrotizing lesion in the left lower extremity, which was later covered with a skin graft. (B) Lower extremity injuries were treated conservatively with dressings and closure by second intention.

The lesion extended to the dorsum of the hand during hospitalization. Doppler ultrasound revealed cellulitis (without collections), while video-capillaroscopy indicated deep capillary thrombosis, leading to the initiation of enoxaparin treatment. Pathological examination revealed ischemic necrosis and suppurative panniculitis associated with microvascular thrombosis (see Figure 2). In paraclinical studies, severe thrombocytopenia, elevated fibrinogen, D-dimer levels, and prolonged coagulation times were noted. Due to prolonged coagulation times and refractory thrombocytopenia despite serial platelet transfusions, the surgical procedure was deferred, and wound care and closure by secondary intention were performed (see Figure 3). The immunology department indicated IVIG therapy due to the low IgG levels, reported as 272 mg/dL (see Table 1).

**Table 1.** Clinical laboratories.

	Arrival	Discharge	
RBC	3.61	4.27	M/uL
HGB	9.5	10.8	g/dL
HCT	29.2	33.1	%
MCV	80	77	fL
MCH	26.3	25.3	Pg
MCHC	32.5	32.7	g/dL
RDW	23.2	13.6	%
WBC	12.9	12.9	K/uL
NEU	9.65	7.27	K/uL
NEU%	74.6	56.4	%
LYM	2.03	3.97	K/uL
LYM%	15.7	30.8	%
MONO	1.09	1.04	K/uL
MONO%	8.4	8.07	%
EOS	0.003	0.470	K/uL
EOS%	0.02	3.64	%
BASO	0.17	0.135	K/uL
BASO%	1.32	1.05	%
PLT	61.8	71.5	K/uL
MPV	8.2	6.1	fL
	Result		
C3	172	mg/dL	
C4	35	mg/dL	
Antithrombin III	88	%	
C-Reactive Protein	69	mg/L	
S-Protein	46	%	

**Figure 4.**
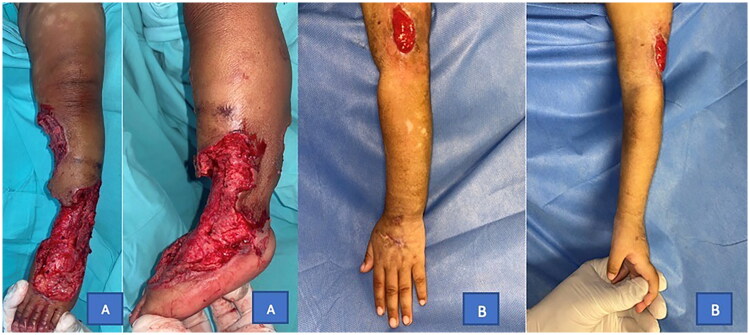
Left lower and upper extremity 6 months postoperatively with completely healed wounds.

Due to the patient’s unusual evolution and the results of the paraclinical studies and pathological studies WAS was suspected. Whole exome sequencing revealed a pathogenic variant in the WAS gene, c.530del (p. Leu177Argfs*84) that generates a truncated protein, confirming the diagnosis. Follow-up examinations revealed no subsequent complications (see Figure 4). The patient continues treatment of his skin lesions with surgical debridement and coverage using antimicrobial dressings and negative pressure therapy on the larger wounds; as well as IVIG therapy and evaluation for a possible bone marrow transplant.

## 3. Discussion

This case is notable for its rarity and the limited literature available on similar presentations regarding the skin. It describes a patient with thrombocytopenia and bleeding, including petechiae that ­progressed to necrotizing skin lesions—an evolution that is seldom reported. Typically, acute presentations involve petechiae, bruising, and bloody diarrhea. Excessive bleeding during circumcision is also common and can serve as an early diagnostic clue. In this case, the absence of the characteristic bite mark often seen in the initial days following Loxosceles envenomation was striking. Additionally, the presence of thrombocytopenia at admission—an atypical finding not usually associated with Loxosceles venom—prompted a broader differential diagnosis to identify alternative etiologies that could explain both the bleeding and skin findings.

Eczema occurs in about 80% of males with Wiskott-Aldrich syndrome. Infections, such as suppurative otitis media, bacterial pneumonia, and dermatologic infections, are frequent complications, potentially explaining the severity and outcome in our case. Approximately 50% of patients with WAS develop food allergies along with ecczema. Treatment typically involves the use of emollients and steroids, with topical tacrolimus often utilized as a steroid-sparing agent. In severe cases, specialist input from dermatology is warranted [8,9].

In our patient’s case, besides receiving specific immunodeficiency therapy and multiple blood transfusions to improve thrombocytopenia and prolonged coagulation times, treatment for necrotizing lesions on the extremities was administered. The diagnostic approach involves flow cytometry with the addition of WASP and WAS gene sequencing. Abnormal WASP expression typically correlates with variations in disease severity. Inconclusive diagnoses warrant WAS gene sequencing [10].

As described in the literature, debridement of necrotic tissue and subsequent skin grafting were performed on the leg lesion. For the upper limb lesion, any surgical intervention was deferred due to the patient’s hematologic conditions: refractory thrombocytopenia and prolonged coagulation times despite blood transfusions. Debridement and wound care were conducted to achieve healing by secondary intention.

Medical treatment should target the patient’s present ailments. For recurrent viral, bacterial, and fungal infections, intravenous immunoglobulin therapy is recommended, along with prophylactic antibiotics for bacteria like *Pneumocystis jiroveci*, and case-by-case evaluation for fungal and viral infections [4].

Severe autoimmune disease presentations may benefit from immunomodulatory therapy, including intravenous immunoglobulin, while corticosteroids are commonly used, albeit with adverse effects monitoring [11]. Hematopoietic stem cell transplantation (HSCT) is the potentially curative treatment for SWA patients, considering their life-threatening conditions. Every SWA-diagnosed patient should initiate curative HSCT treatment [12].

This case report is limited by the absence of parental DNA sequencing and WAS protein expression by flow cytometry. These tests were not performed due to resource constraints and the urgency of initiating treatment. Nonetheless, the diagnosis was supported by clinical findings, immunologic profile, and identification of a known pathogenic *WAS* gene variant.

The true intellectual challenge in identifying the aetiology of a dermatological condition, with non-specific clinical manifestations and a wide spectrum of diagnostic possibilities, lies in discerning the most common causes to the least probable, and within these, autoimmune origins and those less frequently such as syndromic ones. To have an appropriate and early diagnosis, involving the proper specialists might be the most important factor in completing the diagnosis process.

## 4. Conclusion

Wiskott-Aldrich Syndrome (WAS) is a rare primary immunodeficiency with complex and often underrecognized clinical manifestations. This case highlights the limited understanding that persists regarding its early presentation, particularly in the context of ­atypical cutaneous findings. Although uncommon, autoimmune disorders such as WAS should be included in the differential diagnosis when evaluating skin lesions that deviate from the expected clinical course. Moreover, prompt surgical intervention in cases of necrotizing lesions is essential to reduce the risk of secondary infections. Sharing rare clinical presentations of WAS is crucial to enhance awareness, improve diagnostic accuracy, and support timely management by healthcare professionals.
